# Evolution of Intrinsic Disorder in Protein Loops

**DOI:** 10.3390/life13102055

**Published:** 2023-10-14

**Authors:** Fizza Mughal, Gustavo Caetano-Anollés

**Affiliations:** 1 Evolutionary Bioinformatics Laboratory, Department of Crop Sciences, University of Illinois, Urbana, IL 61801, USA; 2 C.R. Woese Institute for Genomic Biology, University of Illinois, Urbana, IL 61801, USA

**Keywords:** chronology, early evolution, flexibility, intrinsically disordered region, loop prototype, molecular function, protein evolution, protein structure, structural domain

## Abstract

Intrinsic disorder accounts for the flexibility of protein loops, molecular building blocks that are largely responsible for the processes and molecular functions of the living world. While loops likely represent early structural forms that served as intermediates in the emergence of protein structural domains, their origin and evolution remain poorly understood. Here, we conduct a phylogenomic survey of disorder in loop prototypes sourced from the ArchDB classification. Tracing prototypes associated with protein fold families along an evolutionary chronology revealed that ancient prototypes tended to be more disordered than their derived counterparts, with ordered prototypes developing later in evolution. This highlights the central evolutionary role of disorder and flexibility. While mean disorder increased with time, a minority of ordered prototypes exist that emerged early in evolutionary history, possibly driven by the need to preserve specific molecular functions. We also revealed the percolation of evolutionary constraints from higher to lower levels of organization. Percolation resulted in trade-offs between flexibility and rigidity that impacted prototype structure and geometry. Our findings provide a deep evolutionary view of the link between structure, disorder, flexibility, and function, as well as insights into the evolutionary role of intrinsic disorder in loops and their contribution to protein structure and function.

## 1. Introduction

Intrinsically disordered regions (IDRs) are functionally important regions of proteins that lack stable structural integrity, are abundant in eukaryotic and viral proteomes, and are widely present in archaea and bacteria [1]. In addition to their deviant sequence behavior, IDRs display distinctive biophysical properties in terms of their sequential, structural, and spatiotemporal heterogeneity to qualify as ‘edge of chaos’ systems [2]. The flexibility of IDRs due to such heterogenous properties endows them with a functional advantage over their structured counterparts that enables their participation in complex biological functions, such as recognition, regulation, and signaling [3]. However, the behavior of these ‘edge of chaos’ systems is sensitive to environmental perturbations and mutations that can lead to misidentification and mis-signaling. Such dysfunction of IDRs has been observed to play a role in amyloidosis, cancer, cardiovascular disorders, and neurodegenerative diseases [3]. Therefore, evolutionary forces act on such ‘non- regular secondary structure regions’ to preserve biological function [4,5].

Remarkably, IDR dynamic behavior is evolutionarily conserved, despite low sequence conservation [5]. Furthermore, flexibility is conserved in proteins [6]. Thus, measuring intrinsic disorder can quantify the inherent flexibility of proteins. Protein loops, the major contributors to structural flexibility, are a source of functional heterogeneity and are therefore important to understanding the relationship between function and flexibility [7,8]. Furthermore, the functional activities of proteins have been proposed to be determined by the molecular functions of loops known as ‘elementary functional loops’ (EFLs) [9]. The EFLs are enriched in amino acid residues responsible for a specific function, with abundant sets of prototypes, including the p-loop prototype responsible for a majority of enzymatic functions [10]. EFLs have proven useful in studying the evolution of protein function in archaeal organisms, suggesting that the use of loop classification systems is a promising route to understanding functional innovation by the reuse of such components in different molecular contexts [11]. In fact, coupling EFLs with network science has provided evolutionary insights into the formation of complex protein structures through the recruitment of loops [12]. Disorder in proteins has been extensively studied at the proteomic level [13,14,15,16] and to a limited extent at the protein structural domain level [17]. However, disorder in loop structures, one of the most granular levels of the hierarchy of molecular structure, remains the least explored despite being fundamental contributors to the flexibility of proteins.

A previous exploration of contact order in proteins, which is correlated to structural flexibility, showed that there are important evolutionary constraints acting on folding speed [18]. It showed that folding speed increases in evolution. An evolutionary study of loops with network approaches that traced the birth of structural domains from loop structures has been conducted in a separate study [19]. Here, we investigate the evolution of disorder at the protein loop level, one of the lowest levels of organization in biological molecular systems. We surveyed thousands of loop prototypes derived from ArchDB [20] and traced their evolutionary history by mapping them to the history of the corresponding structural domains defined at the fold family (FF) level of structural abstraction of the Structural Classification of Proteins (SCOP) [21]. This evolutionary history is based on reliable phylogenomic reconstruction methods that are relatively robust with high mutation rates, horizontal gene transfer, and genetic mosaicism when compared to traditional sequence methods [22].

## 2. Materials and Methods

**Figure 1:**
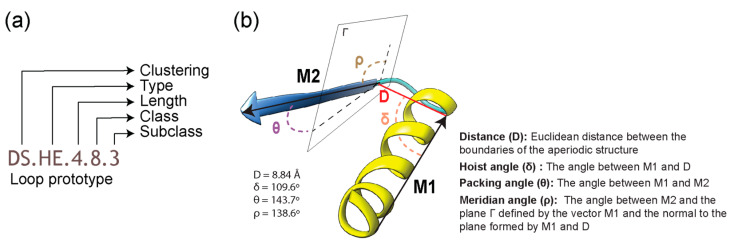
Definition of a loop in ArchDB [19]. (**a**) Classification hierarchy denoted by a loop prototype designation. The prototype is defined by the clustering method used, the bracing secondary structures of the loop (type), the number of residues forming the aperiodic structure, its conformation (ϕ and ψ backbone dihedral angles of the participating residues), and the geometry of the loop. Refer to Table 1 for structural “type” categories. (**b**) Geometric properties of a loop are given by a distance between the boundaries of bracing secondary structures (D) and delta (hoist), theta (packing), and rho (meridian) angles. For illustration, properties are annotated on the 4ETP_A_448 loop structure belonging to the DS.HE.4.8.3 prototype.

We performed intrinsic disorder analysis of loop structures associated with loop prototypes classified by the ArchDB database [20]. Loop prototypes (Figure 1) define the ArchDB classification based on a set of geometric properties, with the following naming scheme (Figure 1a): clustering method used, ‘type’ of bracing secondary structures (Table 1), length of the unstructured loop region between the bracing secondary structures, class, and subclass [20]. Two types of clustering methods have been used for classification in ArchDB: Density Search (DS) and Markov Clustering (MCL). Both methods classify loop lengths differently. The DS algorithm is stringent with the classification of loops because it allows only a fixed ‘length’ of loops to be grouped together, while MCL allows for variation. A class clusters loops with the same conformation of the loop region, while a subclass groups loops with a common geometry (Figure 1b).

**Figure 2:**
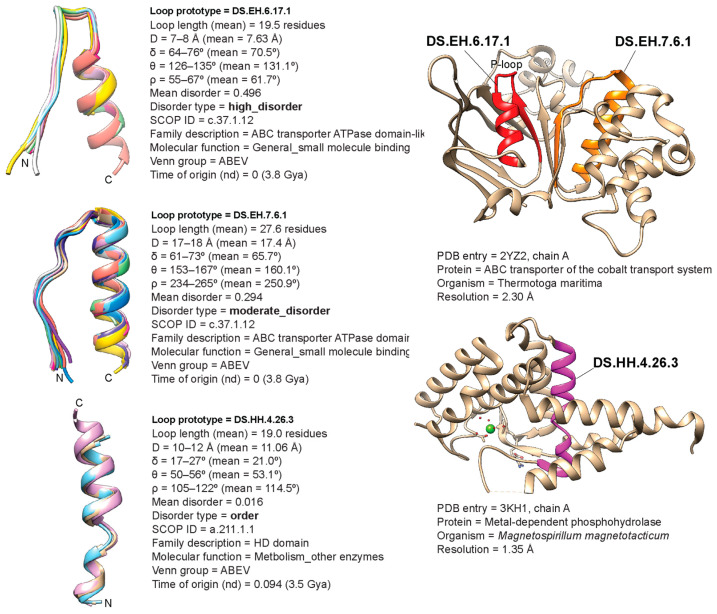
The most ancient loop prototypes in the high disorder, moderate disorder, and order categories (**left**) and their placement in structural domains of modern hydrolase enzymes (**right**).

A loop structure is the region in a protein data bank (PDB) structure annotated with a loop prototype, named by its parent PDB structure, chain, and location of its first residue in the parent structure; e.g., the loop in Figure 1b is part of chain A of PDB entry 4ETP, beginning at residue 448. Several loop structures make up a structural domain in a protein (Figure 2). Domains are the structural, functional, and evolutionary units of proteins. They are defined at the FF level of SCOP classification.

The loop structural dataset of ArchDB [20] holds 190,573 classified loop structures out of a total of 306,726 reported loops. The dataset associated with Density Search (DS) loop prototypes, which holds 125,824 loops, was filtered using mappings of FFs to loop prototypes at e-value <0.001. This resulted in 88,321 loop structures corresponding to 7110 unique DS prototypes. Note that each loop structure in ArchDB has one loop prototype annotation associated with it for a particular classification system (DS, in our case). However, many-to-many annotations exist between loop prototypes and SCOP FFs. We mapped the SCOP FFs from ArchDB to those in our phylogenomic timeline, followed by retaining the loop prototypes mapped to only one SCOP FF. This resulted in 5125 loop prototypes mapped to 1965 FFs (Table S1). We then transferred the times of origin of SCOP FFs to the associated loop prototypes as previously described [19]. These evolutionary ages were measured as node distances (*nd*) extracted from a published phylogenomic tree reconstructed from a genomic census of FFs in 8127 proteomes belonging to the three superkingdoms of life and viruses [23], using thoroughly tested phylogenomic protocols [24,25]. Cellular organisms were represented by 139 archaeal, 1734 bacterial, and 210 eukaryal proteomes. The virus supergroup was represented by 6044 viral proteomes [26]. Figure S1 describes the general experimental workflow that was utilized to build the published phylogenomic tree and the annotated loop chronologies of this study.

Structural disorder was computed using a local copy of the IUPRED2 software with the ‘short’ disorder option [27]. A residue was categorized as disordered if it scored above a threshold of 0.5. Disorder of a loop structure was calculated as a fraction of the disordered residues to the total number of residues. The mean disorder for each loop prototype was the average of disorder scores for individual loop structures associated with each loop prototype:

$$\mathrm{Mean}\mathrm{disorder}\mathrm{in}a\mathrm{loop}\mathrm{prototype}=\frac{\mathrm{disorder}\mathrm{fraction}\mathrm{of}\mathrm{loop}\mathrm{structures}\mathrm{for}\mathrm{loop}\mathrm{prototype}}{\mathrm{total}\mathrm{number}\mathrm{of}\mathrm{loop}\mathrm{structures}\mathrm{for}\mathrm{loop}\mathrm{prototype}}$$

A loop prototype was classified as ‘ordered’ if its mean disorder score was from 0 to 0.1, ‘moderate disorder’ with a mean disorder score from 0.1 to 0.3, and ‘high disorder’ with scores greater than 0.3.

## 3. Results

We conducted a disorder analysis on 5125 loop prototypes associated with 1965 FFs of structural domains. The FFs were annotated with the times of origin (evolutionary ages given as *nd* values) derived from a genomic census of 8127 proteomes from the three superkingdoms and viruses. The evolutionary ages are based on phylogenomic methods benchmarked by well over a decade of research and experimentation [28,29,30,31,32,33]. We inspected the levels of disorder and various structural and geometric properties of loop prototypes in superkingdoms and viruses, indexed their associated molecular functions, and explored the evolutionary spread of prototypes in a phylogenomic timeline.

Figure 2 shows the structural alignments of the oldest loop prototypes of the high disorder, moderate disorder, and order categories of proteins, illustrating the type of data gathered in our analysis. For each loop prototype entry, we indexed a set of geometric properties, mean disorder values, an index of disorder types, family and molecular function annotations, distribution in life, and the time of origin of the loop structure (Table S1). Figure 2 also illustrates the tracing of loop structures onto their corresponding structural domains using hydrolase enzymes as scaffolds.

**Figure 3:**
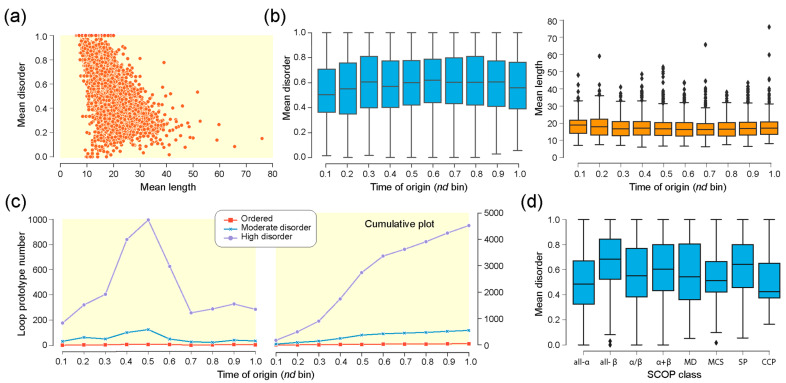
Disorder in loop prototypes. (**a**) Mean disorder scores plotted against mean length of 5125 protein loop prototypes. Correlations were significant (Spearman’s correlation coefficient = −0.736, *p*-value = 0). (**b**) Mean disorder and mean loop length of prototypes binned by times of origin measured as evolutionary age (*nd*) of corresponding SCOP fold families (FFs). The box-and-whisker plots display 5-number data summaries (minimum, first quartile, median, third quartile, and maximum) and data outliers (rhomboids). (**c**) Counts and cumulative counts of loop prototypes introduced via associated SCOP FFs in time represented by bins of evolutionary age (*nd*). (**d**) Mean disorder of loops mapping to domain in SCOP classes.

There appears to be a sharp decline in mean disorder scores with an increase in mean loop structure length (Figure 3a). However, while the median of mean disorder scores gradually increased with time of origin, the median of the mean length of the loop structure was steady throughout the timeline (Figure 3b). As expected, loops with high disorder outnumbered those with moderate and low disorder as their accumulation rates increased and decreased in the timeline (Figure 3c). Interestingly, the medians of the mean disorder score of loop prototypes in SCOP classes of structural domains (Figure 3d) showed a general increase with age. Following a rejection of the null hypothesis of all medians being the same by the Kruskal–Wallis H test [34], Conover’s test of multiple comparisons [35] indicated that the pairwise comparison of the four major classes of domains, namely, all-α, all-β, α + β, and α/β, showed a significant difference in medians (Table 2). The mean disorder increased according to the sequence: all-α < α/β < α + β < all-β (Figure 3d). Moreover, out of the forty-eight ‘ordered’ loop prototypes, eighteen belonged to FFs from the α/β class, followed by eleven from all-α, seven from α + β, five from all-β, and seven belonging to rest of the classes (Table S2).

**Figure 4:**
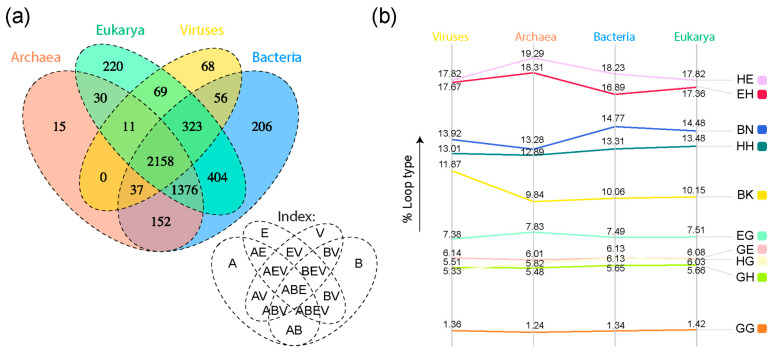
Comparative genomic analysis of loop prototypes. (**a**) Venn diagram of prototypes distributed among superkingdoms of life and viruses. Venn groups describe the distribution of prototypes in supergroups Archaea (A), Bacteria (B), Eukarya (E), and Viruses (V). Their names are indexed below the diagram. Prototypes shared by all supergroups belong to the ABEV Venn category; those shared by three supergroups (e.g., ABE prototypes shared by all cellular superkingdoms) belong to either ABE, BEV, ABV, or AEV; those shared by two supergroups belong to BE, AB, AE, BV, or EV; and those unique to supergroups belong to A, B, E, or V. Taxonomic groups are mapped to loop prototypes through the SCOP FFs they belong to. (**b**) Slope graph describing percentages of each loop type in each superkingdom and viral supergroup.

A four-set Venn diagram describing the distribution of loop prototypes in supergroups, i.e., superkingdoms Archaea (A), Bacteria (B), and Eukarya (E) and Viruses (V), showed a high number of loop prototypes present in all supergroups (the ABEV Venn group) and in all superkingdoms (the ABE Venn group) (Figure 4a). The α-β type ‘HE’ claimed the highest percentages of loop types present in each superkingdom and the viral supergroup (Figure 4b). The distribution of loop types appeared to follow a similar trend for all superkingdoms and viruses.

**Figure 5:**
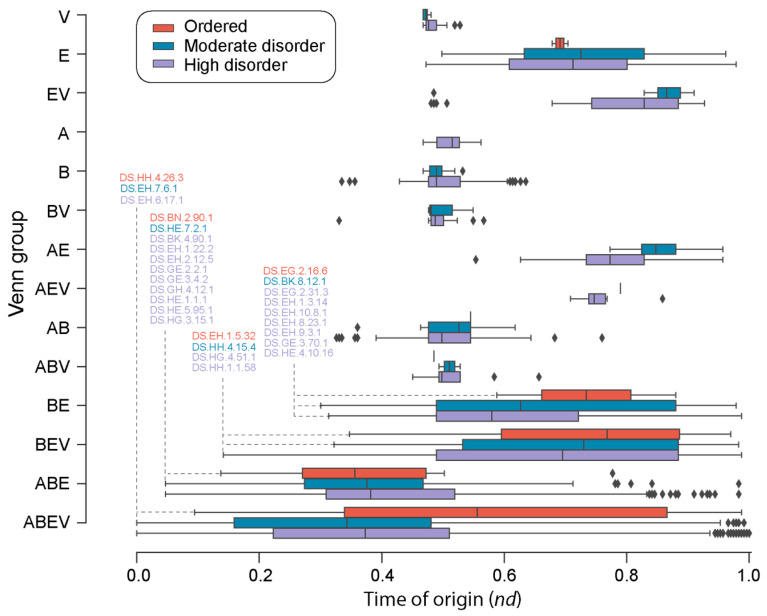
A chronology of loop prototypes categorized by Venn group and magnitude of disorder. The time of origin of each prototype is given as an evolutionary age measured in node distance (*nd*) units. Venn groups describe the distribution of prototypes in supergroups Archaea (A), Bacteria (B), Eukarya (E), and Viruses (V) following nomenclature defined in Figure 4a. Note that the AV Venn group is absent (see Figure 4a). First prototypes appearing in each disorder category are annotated for the ABEV, ABE, BEV, and BE Venn groups.

A closer inspection of the distribution of loop prototypes belonging to each Venn group (Figure 5) along the evolutionary timeline revealed patterns of first origin matching those observed for the FFs. As a general trend, ‘ordered’ prototypes (Table S2) appeared later than high- and moderate-disorder prototypes, with 39 ‘ordered’ prototypes appearing around and after *nd* = 0.4. Highly and moderately disordered prototypes appeared concurrently (roughly at the same time) for the ABEV, ABE, EV, and V Venn groups. However, moderately disordered prototypes appeared earlier than highly disordered ones in the FFs of the BE group. Interestingly, the A Venn group had only high-disorder loop prototypes. Evolutionary tracings also showed that while high-disorder prototypes were present in all Venn groups (except AV), twenty-four of the forty-eight ‘ordered’ prototypes were only present in the FFs of the ABEV group, followed by thirteen present in ABE, four in BEV, two in AB, two in BE, two in E, and one in ABV (Table S2).

**Figure 6:**
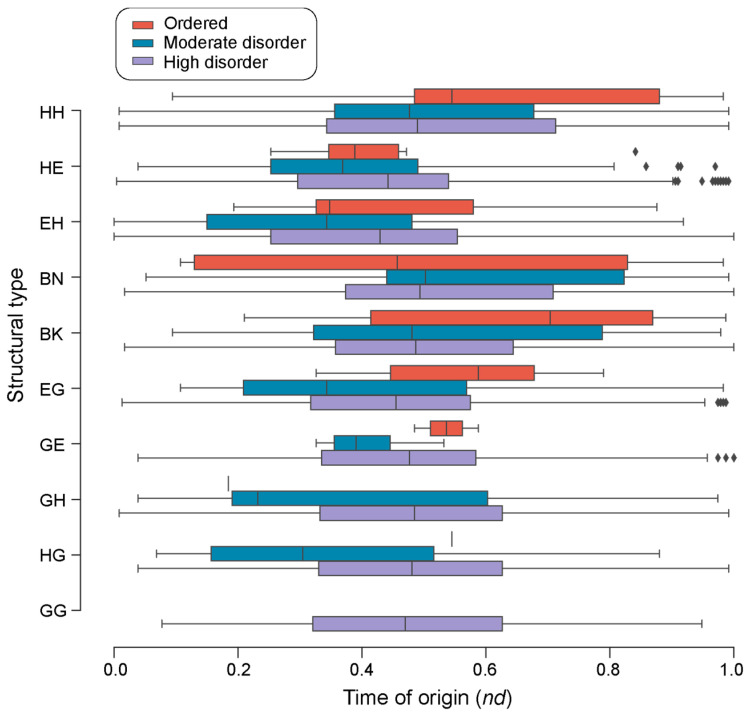
A chronology of loop prototypes categorized by structural types and magnitude of disorder. The time of origin of each prototype is given as an evolutionary age measured in node distance (*nd*) units.

The distribution of loop structural ‘types’ along the evolutionary timeline showed that all types appeared very early in evolution. The ‘DS.EH.6.17.1’ and ‘DS.EH.7.6.1’ prototypes appeared the earliest together with the most ancient ‘ABC transporter ATPase domain-like’ FF (c.37.1.12) (Figure 5 and Figure 6). Highly and moderately disordered prototype types α-α (HH) and β-α (EH) appeared approximately at the same time. Except for the helix 3_10_-helix 3_10_ (GG) prototypes, all other ‘types’ had both ordered and moderately disordered prototypes. For the remaining seven types, highly ordered prototypes appeared earlier than both moderately disordered and ordered prototypes. There were thirteen ‘ordered’ prototypes associated with the HH type, followed by nine with EH, seven with BK, six with HE, five with EG, four with BN, two with GE, and one each with HG and GH.

**Figure 7:**
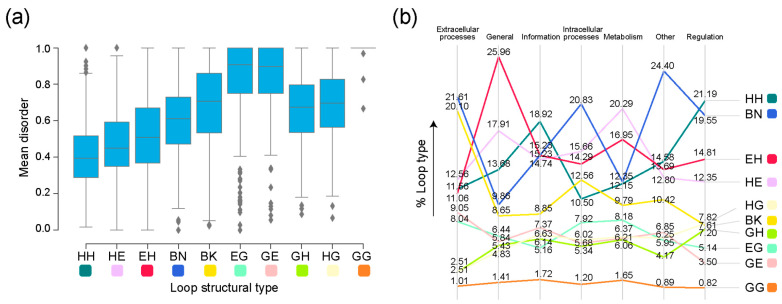
Disorder in loop prototypes grouped on the basis of molecular function. (**a**) Distribution of mean disorder scores by loop type. Loop types are described in Table 1. (**b**) Slope graph describing percentages of each loop type in each molecular function in the sampled dataset. Molecular functions are mapped to loop architectures through their corresponding SCOP FFs.

The median values for mean disorder scores by structural type were the highest for the helix 3_10_-containing GG, EG, and GE prototypes, with a left skew in their respective distributions (Figure 7a). To assess whether higher disorder scores for specific types were associated with the molecular function of the FFs they belong to, we inspected the distribution of structural types for each molecular function (Figure 7b). Some of the functional categories showed a preference for certain types of prototypes. The β-β hairpin (BN) type comprised the highest number of prototypes present in FFs belonging to the ‘Intracellular processes’, ‘Extracellular processes’, and ‘Other’ categories. The α-α (HH) type dominated the distribution in FFs in both ‘Information’ and ‘Regulation’. The FFs associated with the ‘General’ and ‘Metabolism’ functional categories were associated with a high number of EH and HE types, respectively.

**Figure 8:**
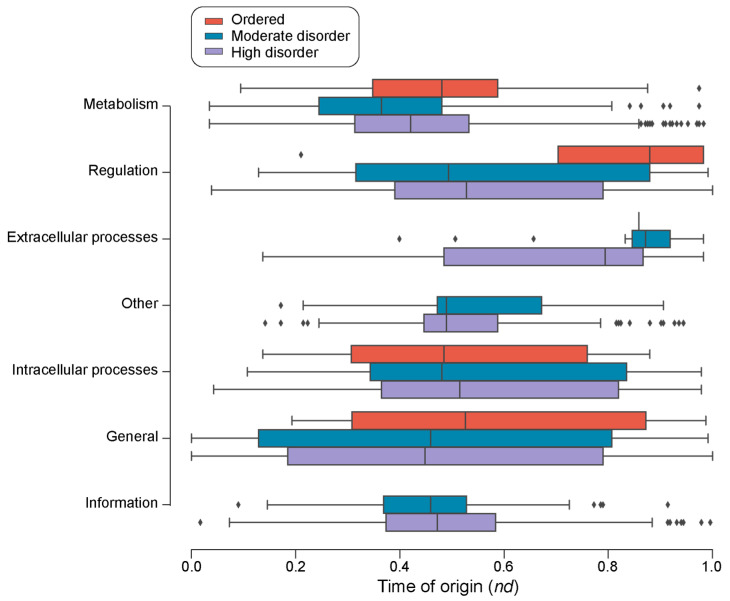
A chronology of loop prototypes categorized by molecular function and magnitude of disorder. The time of origin of each prototype is given as an evolutionary age measured in node distance (*nd*) units.

The survey of loop prototypes by disorder categories in molecular function showed that ‘Information’, ‘Extracellular processes’, and ‘Other’ were the only categories with no associated ‘ordered’ prototypes (Figure 8). Out of the forty-eight ‘ordered’ prototypes, twenty-nine belonged to ‘Metabolism’ FFs, followed by seven to ‘Intracellular processes’ FFs, six to ‘General’ FFs, five to ‘Regulation’ FFs, and one to ‘Extracellular processes’ FFs (Table 3). A Gene Ontology (GO) enrichment analysis of the FFs with ordered prototypes showed that these FFs are highly enriched in activities mainly related to metabolism, transport, and DNA transcription as well as pathogenesis and immune response (Table 3). Highly and moderately disordered prototypes appeared approximately at the same time in the FFs belonging to ‘Metabolism’ and ‘General’ (Figure 8). For FFs in the other functional categories, highly disordered prototypes appeared earlier than moderately disordered prototypes, and both appeared earlier than ordered prototypes when these were present.

**Figure 9:**
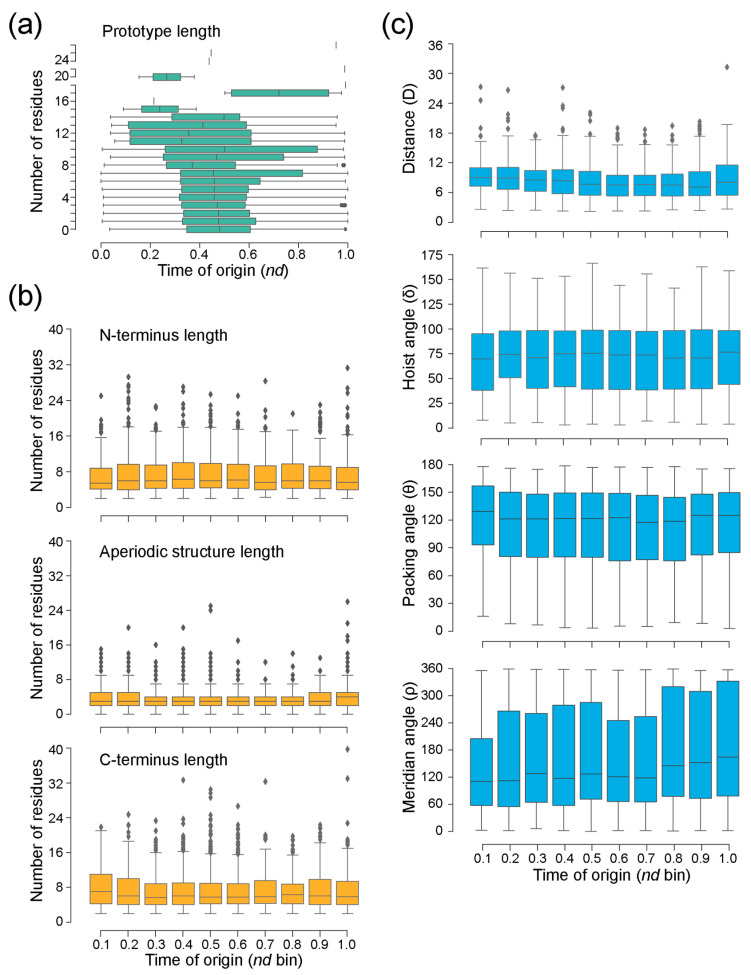
An evolutionary chronology of the structural properties of loop prototypes. The time of origin of each prototype is given as an evolutionary age measured in node distance (*nd*) units. (**a**) Distribution of loop lengths along the evolutionary timeline. (**b**) Distribution of loop length, average N-terminus and C-terminus lengths in an architecture along the binned evolutionary timeline. Note that ArchDB classification has loop lengths of ‘zero’ that represent architectures with no aperiodic residues, representing shifts and transitions between different secondary structures [19]. (**c**) Distribution of average geometric measures of structures in a loop architecture in evolutionary time. Geometric measures include distance and delta (hoist), theta (packing), and rho (meridian) angles (Figure 1b).

Loop prototypes with smaller lengths of the loop region, ranging 1–7, were widespread throughout evolutionary time, while longer prototypes were part of FFs that appeared relatively late in evolution (Figure 9a). The average length for N-terminus and C-terminus of prototypes showed consistent distribution with little variation throughout the timeline. Similarly, geometric properties of prototypes, namely, hoist (delta) and packing (theta) angles, and distance were spread consistently throughout evolutionary time. However, the median values for meridian (rho) angles showed an increase with time, while the Euclidean distance (D) between the boundaries of aperiodic structures showed a slight decrease (Figure 9b).

## 4. Discussion

Protein loops constitute a diverse group of molecular building blocks made of helix, strand, and coil segments that are largely responsible for the processes and molecular functions of the living world [37]. They can interact with solvents, ligands, and other molecules, establishing a wide range of dynamic behaviors, from static to highly plastic. From an evolutionary perspective, loops likely represent prior structural states of the type envisioned by Margaret O. Dayhoff [38,39] more than half a century ago, intermediate evolutionary forms responsible for the emergence of structural domains in proteins [19]. The flexibility of protein loops likely reflects this early evolutionary start from short peptides made of simplified amino acid sequences [10,11,12], frequently glycine-rich [40]. The evolutionary introduction of more complex and ordered structures altered the initial order–disorder balance to fine-tune protein function, as recently shown with allosteric regulation [41] and the divergence of allosteric communication in major folds and repeat proteins [42]. Such evolutionary fine-tuning can help engineering and de novo design efforts that search for new or modified protein functions.

Here, we study loop flexibility by surveying intrinsic disorder and following its evolution. We focus on loop prototypes, supersecondary motifs bracing nonregular aperiodic regions sourced from the ArchDB database, a library that has sampled all loop geometries and should be considered essentially complete [43]. Two types of prototypes exist, those that are modular and are recruited throughout evolution and those that are non-modular and are recruited into domains only once [19]. To offset the confounding effects of recruitment, we here focus on the latter. Our analysis revealed the central evolutionary role of disorder and flexibility, the unexpected evolutionary rise of structural order, and evolutionary constraints percolating from higher to lower levels of biological organization leading to trade-offs between flexibility and rigidity as well as impacts on the structure and geometry of prototypes.

### 4.1. The Evolutionary Centrality of Loop Disorder and Flexibility

We found that loop prototypes of ancient FFs were always more disordered than their derived counterparts and that ordered prototypes developed later in evolution. These patterns were present when indexing timelines with Venn groups of prototype distributions in superkingdoms and viruses (Figure 5), types of bracing secondary structures (Figure 6), or annotated molecular functions (Figure 8). Furthermore, we found a trend of mean disorder increasing in evolution (Figure 3b), with disorder being widely present in prototypes throughout the timeline (Figure 3c). This was an expected outcome given that flexibility and intrinsic disorder are linked phenomena and that protein folding speed is correlated with flexibility and is evolutionarily optimized to increase over time [18]. However, significant differences in mean disorder distributions of the four major SCOP classes, namely, the α/β, α + β, all-α, and all-β proteins, were detected (Figure 3d), suggesting there are evolutionary constraints acting both across levels of biological organization and within systems. This is by no means surprising. SCOP classes are known to harbor deep phylogenomic signatures in their makeup (reviewed in [44]).

### 4.2. The Unexpected Evolutionary Rise of Order in Loop Structure

Remarkably, our analysis reveals the presence of a significant number of ‘ordered’ loop prototypes (Table S2) developing later than disordered prototypes but quite early in evolutionary history (Figure 5). Figure 2 describes the oldest ordered prototype, DS.HH.4.26.3, which can be found embedded in the HD-domain FF of a metal-dependent phosphohydrolase. The conserved amino acid residues of these enzymes are enriched in histidine and aspartate. The existence of ordered loops poses the question of the purpose of their existence. A potential explanation is the preservation of molecular functions that require a certain amount of structural integrity, which then becomes evolutionarily ‘canalized’ into conserved regions. As an example, consider two RIG-I-like receptors (RLRs), RIG-I and MDA5, which play a vital role in vertebrate antiviral defense [45]. Both receptors share high sequence similarity but perform nonredundant functions, as each receptor recognizes different types of double-stranded RNA (dsRNA) viruses [46]. Differential flexibility of a loop that is rigid in RIG-I, but highly disordered in MDA5, enables each receptor to perform its respective sensory functions. Remarkably, we see an enrichment of immune response biological processes in ordered prototypes (Table 3). Similarly, pathogenesis-related class-10 (PR10) proteins, found in plants in response to stress-inducing factors, are hypothesized to play a role in defense against plant pathogens [46]. The PR10 proteins possess a glycine-rich L4 loop, similar to the highly flexible P-loop present in many proteins. However, the L4 loop is found to have unusual rigidity, where it differs from the P-loop, despite its high glycine content [47].

### 4.3. Percolation of Evolutionary Constraints from Higher to Lower Levels of Organization

Eukaryotic and viral proteomes have relatively greater disorder than those belonging to archaea and bacteria [14]. To dissect patterns of sharing and the spread of high disorder in loop prototypes, we analyzed their association to FFs in superkingdoms and viruses. The presence of all structural types of prototypes in the ancient and universal core shared by all life (the ABEV Venn group), different patterns of prototype accumulation in Venn groups of superkingdoms and viruses (Figure 4), and patterns of accumulation along the evolutionary chronology of prototypes (Figure 5) has important implications to our understanding of the evolution of intrinsic disorder. First, the findings suggests that all ‘types’ of prototypes were abundant in ancient cells and viruses. A wide variety of prototype building blocks were therefore available in the early protein world to make more complex structures. Second, over time, some lineages appear to have lost certain types of prototypes that were not useful to them. Instead, they favored and retained prototypes that provided them with an evolutionary advantage, most likely in terms of survival and reproduction. Indeed, short disordered regions in the ‘context’ of a protein enable or complement the function of a structured domain and sometimes act as separate functional modules [48]. Remarkably, the absence of certain structural types of prototypes in the A, ABV, AEV, BV, and EV Venn groups coincides with the branching of major organismal lineages and viruses. It also suggests that evolutionary constraints at higher proteomic and structural domain levels are percolating at lower levels to impact the evolutionary spread of disorder. Likewise, ‘ordered’ prototypes, which developed later in time, serve different purposes in prokaryotic microbes and viruses. Only prototypes shared by all life (ABEV), shared by cellular organisms (ABE), and shared by organisms with the same membrane phospholipid makeup (BE and BEV) developed ordered structures and did so late in the evolution of their respective groups (Figure 5). Conversely, ordered prototypes were almost absent in prototypes specific to superkingdoms and viruses. In fact, only highly disordered prototypes were specific to Archaea, and most virus-specific and bacteria-specific prototypes showed only high or moderate disorder. This differential behavior appears to hold a strong historical signature that is indicative of constraints percolating from the organismal level to the loop structural level.

### 4.4. Evolutionary Percolation and Trade-Offs between Flexibility and Rigidity in Loop Behavior

We note that there is considerable variation in the distribution of disorder in all ten structural types of loop prototypes (Figure 7). Flexibility is critical to the functioning of proteins [49]. It is therefore expected that the differential flexibility of types of prototypes will be differentially adopted by categories of molecular function. Indeed, some functional categories prefer loops bearing certain structural types (Figure 7). For example, prototypes associated with ‘Metabolism’ and ‘General’ possessed a high number of ordered prototypes (Figure 8; Table S2). This observation can be reconciled with the finding that enzymes require a balance of structural rigidity and flexibility in order to carry out their functions [50]. High flexibility may lead to conformational states that hinder enzymatic activity, interfering with enzyme–substrate interactions. Conversely, FFs from ‘Extracellular processes’ have a greater number of flexible loops of types β-β hairpin (BN) and β-β link (BK). The FFs of the sampled prototypes associated with ‘Extracellular processes’ are mostly associated with immune response, toxins and defense enzymes, and cell adhesion. Viruses have evolved disorder as means of evading host immune responses and for mimicking host functions [51,52]. This variation in disorder for different functional categories also indicates varying speeds of the evolutionary clock, depending on the nature of the function [53]. Note that prototype disorder in extracellular processes might not only reflect virus evolution, but also the dynamic adaptation of hosts to viruses or other pathogens, especially those interacting with eukaryotes.

The early evolutionary rise of high levels of disorder in loop prototypes contrasts with the evolution of disorder in the structural domains of proteins. A parallel analysis of intrinsic disorder in ~3800 FFs that were present in the same 8127 proteomes of superkingdoms and viruses examined in this study revealed ancient FFs were ordered and that disorder of structural domains evolved as a benefit acquired later in evolution (Mughal and Caetano-Anollés, ms. in preparation). Thus, loop-associated ‘short’ and domain-associated ‘long’ regions of disorder evolve differently across different levels of protein organization. We note that different evolutionary constraints acting at different levels of biological complexity are also observed in evolving metabolic networks where higher and lower levels of metabolic organization are under stringent evolutionary constraints, while the intermediate levels add ‘noise’, thus driving innovation holistically [54]. In fact, the prototypes of the most ancient structural domain, the ABC transporter ATPase domain-like FF (c.37.1.12), illustrates this interplay of constraints. The highly ordered structure of the c.37.1.12 FF (Figure 2) harbors the highly flexible and glycine-rich, phosphate-binding loop, the ‘P-loop’ [55], which we recently showed catalyzes convergence towards a folded structure of the emerging domain [19]. This suggests that evolutionary constraints requiring both relatively rigid domains and highly disordered loops are necessary to carry out function. Thus, evolutionary constraints percolate from the structural domain level to the loop structural level.

Long disordered regions are context-dependent such that their disorder-to-order conformations depend on the presence of specific binding partners or environmental elements, such as pH, redox potential, or temperature [56,57]. Similarly, short disorder regions would be expected to be context-dependent in terms of flanking structures and protein contacts. In fact, short disordered regions exhibit behavior similar to that of regular secondary structure and are more resilient to mutations when compared to regions of long disorder that are highly sensitive to mutations, demonstrating that in contrast with short disorder, maintaining long disorder is evolutionarily nontrivial [58].

### 4.5. Evolutionary Impact on Loop Structure and Geometry

Finally, tracing the history of the structural and geometric properties of loop prototypes along the evolutionary timeline provides additional insights (Figure 9). Loop length appeared to be the major source of evolutionary variability when compared to the lengths of N- and C-terminal bracing loop structures. This shows that flexible regions of proteins are indeed chiefly responsible for functional heterogeneity [8]. There was also slight variation in the hoist (delta) and packing (theta) angles and distance measures of the prototypes along the timeline. Packing density is correlated to evolutionary rates at the protein level, but it varies slightly in evolution in the case of protein loops [59]. This is suggestive of evolutionary constraints acting to maintain hoist and packing in protein fragments with little variation, whilst introducing novelty by varying meridian (rho) angles.

## 5. Conclusions

Our study does not exclusively address intrinsic disorder. Instead, it focuses on studying disorder as a proxy for surveying protein flexibility, an approach taken by other recent studies [47]. The results provide a deeper evolutionary view of the link between structure, disorder, flexibility, and function. First, ancient loop prototypes tended to be more disordered than their derived counterparts, with ordered prototypes developing later in evolution. This highlights the central evolutionary role of disorder and flexibility. Second, there was an unexpected emergence of ordered prototypes early in evolutionary history, possibly driven by the need to preserve specific molecular functions. Third, the study uncovered the percolation of evolutionary constraints from higher to lower levels of biological organization. This percolation influenced the spread of disorder in prototypes. Fourth, the analysis revealed trade-offs between flexibility and rigidity in loop behavior, with different functional categories preferring specific structural types. Finally, tracing the evolution of the structural and geometric properties of loops revealed variations in loop length and geometry along the evolutionary chronology of prototypes. These findings provide valuable insights into the role of protein loops in evolution and their contribution to protein structure and function. Findings also highlight the central evolutionary role of protein folding, which is linked to protein flexibility [60].

We conclude by acknowledging some limitations of our study. First, the accuracy of the disorder analysis relies on the precision of the available software, which introduces the possibility of false negatives and false positives in our analyses. Second, biases within databases, such as the presence of disordered structures in the PDB and, consequently, in ArchDB, may also act as limiting factors. Lastly, there are many-to-many mappings between loop prototypes and FFs with varying degrees of e-values in ArchDB. In our study, we opted for a stringent e-value of <0.001, resulting in prototypes being mapped to only one FF at this e-value. While this choice may lead to missing some hits, it helps mitigate issues that could arise from a high number of false positives. In the future, addressing these limitations can be achieved by expanding the database knowledge and enhancing prediction software accuracy.

## Figures and Tables

**Table 1:** Structural types of loop prototypes in ArchDB [19].

Type	Bracing Secondary Structure
HH	alpha-alpha
HE	alpha-beta
EH	beta-alpha
BN	beta-beta hairpin
BK	beta-beta link
EG	beta-helix 3_10_
GE	helix 3_10_-beta
GH	helix 3_10_-helix
HG	helix-helix 3_10_
GG	helix 3_10_-helix 3_10_

**Table 2:** *p*-values from Conover’s test for pairwise comparison (preceded by rejection of null hypothesis with the Kruskal–Wallis test at *p*-value = 6.045 × 10^−45^). CCP, coiled coil proteins; MCS, membrane and cell surface; MD, multi-domain (α and β); SP, small proteins.

SCOP Class	All-α	All-β	α + β	α/β	CCP	MCS	MD	SP
all-α	−1	3.45 × 10^−44^	1.76 × 10^−20^	4.62 × 10^−10^	1	1	0.04501	4.66 × 10^−5^
all-β	3.45 × 10^−44^	−1	1.21 × 10^−8^	4.36 × 10^−24^	0.31539	0.00021	0.00023	1
α + β	1.76 × 10^−20^	1.21 × 10^−8^	−1	0.00038	1	0.32722	1	1
α/β	4.62 × 10^−10^	4.36 × 10^−24^	0.00038	−1	1	1	1	0.48768
CCP	1	0.31539	1	1	−1	1	1	1
MCS	1	0.00021	0.32722	1	1	−1	1	0.35386
MD	0.04501	0.00023	1	1	1	1	−1	1
SP	4.66 × 10^−5^	1	1	0.48768	1	0.35386	1	−1

**Table 3:** Highly enriched GO “biological process” terms in FFs of 48 ‘ordered’ loop prototypes and a probability score equal to 1 in the Structural Domains Annotation Database (SDADB) [36].

GO ID	GO Description
GO:0000082	G1/S transition of mitotic cell cycle
GO:0005975	carbohydrate metabolic process
GO:0006099	tricarboxylic acid cycle
GO:0006260	DNA replication
GO:0006351	transcription, DNA-templated
GO:0006355	regulation of transcription, DNA-templated
GO:0006508	proteolysis
GO:0006511	ubiquitin-dependent protein catabolic process
GO:0006631	fatty acid metabolic process
GO:0006633	fatty acid biosynthetic process
GO:0006955	immune response
GO:0007165	signal transduction
GO:0009058	biosynthetic process
GO:0009186	deoxyribonucleoside diphosphate metabolic process
GO:0009405	pathogenesis
GO:0015696	ammonium transport
GO:0015976	carbon utilization
GO:0019646	aerobic electron transport chain
GO:0030245	cellulose catabolic process
GO:0031388	organic acid phosphorylation
GO:0043401	steroid hormone mediated signaling pathway
GO:0055114	oxidation–reduction process
GO:0072488	ammonium transmembrane transport

## Data Availability

The data presented in this study are openly available in the ArchDB (http://sbi.imim.es/archdb/; accessed on 13 October 2023), SCOP (https://scop.mrc-lmb.cam.ac.uk; accessed on 13 October 2023) and SCOPe (https://scop.berkeley.edu; accessed on 13 October 2023) repositories. Other data and information supporting the findings of this study are available within the article and its Supplementary Materials.

## Supplementary Materials

The following supporting information can be downloaded at: https://www.mdpi.com/article/10.3390/life13102055/s1. Figure S1: General phylogenomic workflow utilized in this study; Table S1: Prototypes and their structural, functional and phylogenomic annotations; Table S2: List of ‘ordered’ loop prototypes mapped to SCOP FFs represented by their SCOP concise classification strings (*ccs*).
