# Supplementary material for: Evolution of Intrinsic Disorder in Protein Loops
Source: Life (Basel). 2023 Oct 14;13(10):2055. doi: 10.3390/life13102055 (PMC10608553; doi:10.3390/life13102055)
Supplement: Supplementary file 1 [file life-13-02055-s001.zip › Table S2.pdf]

# Evolution of Intrinsic Disorder in Protein Loops

**Fizza Mughal [1] and Gustavo Caetano-Anollés [1,2,*]**

**Table S2.** List of 'ordered' loop prototypes mapped to SCOP FFs represented by their SCOP concise classification strings (*ccs*).

| Loop prototype | SCOP *ccs* | Fold family (FF) description | Age (*nd*) | Molecular function | Function description | Venn group |
|---|---|---|---|---|---|---|
| DS.HG.2.1.35 | e.18.1.1 | Nickel-iron hydrogenase, large subunit | 0.545 | Metabolism | Other enzymes | AB |
| DS.HH.8.9.1 | e.18.1.1 | Nickel-iron hydrogenase, large subunit | 0.545 | Metabolism | Other enzymes | AB |
| DS.BK.5.93.1 | d.41.1.1 | CO dehydrogenase molybdo-protein N-domain-like | 0.356 | Metabolism | Electron transfer/transport | ABE |
| DS.BK.6.34.1 | d.145.1.2 | Uridine diphospho-N-Acetylenolpyruvylglucosamine reductase (MurB), N-terminal domain | 0.472 | General | Small molecule binding | ABE |
| DS.BN.2.90.1 | d.127.1.1 | Creatinase/aminopeptidase | 0.137 | Intracellular processes | Proteases, peptidases, and their inhibitors | ABE |
| DS.BN.4.118.1 | b.69.11.1 | Putative isomerase YbhE | 0.777 | Metabolism | Other enzymes | ABE |
| DS.EH.2.19.5 | c.1.12.7 | Phosphoenolpyruvate mutase/Isocitrate lyase-like | 0.335 | Metabolism | Energy production and conversion | ABE |
| DS.EH.3.33.1 | c.55.1.13 | CoaX-like | 0.3266 | Metabolism | Other enzymes | ABE |
| DS.EH.4.88.1 | c.36.1.6 | TK-like Pyr module | 0.193 | General | Small molecule binding | ABE |
| DS.GH.0.1.119 | d.90.1.1 | NADH oxidase/flavin reductase | 0.184 | Metabolism | Electron transfer/transport | ABE |
| DS.HE.11.1.1 | a.102.1.3 | N-acylglucosamine (NAG) epimerase | 0.472 | Metabolism | Carbohydrate transport and metabolism | ABE |
| DS.HE.4.14.5 | c.69.1.21 | PepX catalytic domain-like | 0.421 | Metabolism | Other enzymes | ABE |
| DS.HE.5.27.3 | c.1.9.15 | PP1699/LP2961-like | 0.356 | Metabolism | Other enzymes | ABE |
| DS.HH.4.92.1 | f.21.2.1 | Fumarate reductase respiratory complex cytochrome b subunit, FrdC | 0.502 | Metabolism | Other enzymes | ABE |
| DS.HH.8.6.1 | f.44.1.1 | Ammonium transporter | 0.270 | Intracellular processes | Transport | ABE |
| DS.BK.4.16.1 | e.1.1.1 | Serpins | 0.880 | Intracellular processes | Proteases, peptidases, and their inhibitors | ABEV |
| DS.BK.6.65.1 | d.144.1.6 | APH phosphotransferases | 0.210 | Regulation | Kinases, phosphatases, and inhibitors | ABEV |
| DS.BK.7.1.2 | c.10.2.7 | Ngr ectodomain-like | 0.987 | General | General or several functions | ABEV |
| DS.BN.12.4.1 | c.67.1.1 | AAT-like | 0.107 | Metabolism | Transferases | ABEV |
| DS.BN.8.37.1 | g.37.1.1 | Classic zinc finger, C2H2 | 0.982 | Regulation | DNA-binding (transcription factors) | ABEV |
| DS.EG.2.16.3 | c.66.1.18 | Mycolic acid cyclopropane synthase | 0.326 | Metabolism | Transferases | ABEV |
| DS.EG.2.16.4 | c.66.1.49 | BC2162-like | 0.446 | Metabolism | Transferases | ABEV |
| DS.EG.2.82.1 | c.66.1.42 | AD-003 protein-like | 0.78969957 | Metabolism | Transferases | ABEV |

| | | | | | | |
|---|---|---|---|---|---|---|
| DS.EH.0.1.88 | c.68.1.7 | 1,3-glucuronyltransferase | 0.876 | Metabolism | Transferases | ABEV |
| DS.EH.1.27.1 | c.7.1.2 | R1 subunit of ribonucleotide reductase, C-terminal domain | 0.481 | Metabolism | Other enzymes | ABEV |
| DS.EH.2.9.10 | c.3.1.6 | Thi4-like | 0.579 | General | Small molecule binding | ABEV |
| DS.EH.3.77.1 | c.53.2.1 | beta-carbonic anhydrase, cab | 0.283 | Metabolism | Other enzymes | ABEV |
| DS.EH.4.73.1 | c.69.1.1 | Acetylcholinesterase-like | 0.721 | Metabolism | Other enzymes | ABEV |
| DS.GE.1.33.2 | b.29.1.15 | Trypanosoma sialidase, C-terminal domain | 0.588 | Metabolism | Secondary metabolites biosynthesis, transport, and catabolism | ABEV |
| DS.HE.2.23.2 | b.50.1.2 | Pepsin-like | 0.841 | Intracellular processes | Proteases, peptidases, and their inhibitors | ABEV |
| DS.HE.5.136.1 | d.159.1.3 | Protein serine/threonine phosphatase | 0.343 | Intracellular processes | Proteases, peptidases, and their inhibitors | ABEV |
| DS.HE.5.158.1 | c.36.1.9 | Pyruvate oxidase and decarboxylase PP module | 0.253 | General | Small molecule binding | ABEV |
| DS.HH.1.3.35 | a.138.1.1 | Cytochrome c3-like | 0.532 | Metabolism | Oxidation/reduction | ABEV |
| DS.HH.10.9.1 | a.74.1.1 | Cyclin | 0.983 | Regulation | DNA-binding (transcription factors) | ABEV |
| DS.HH.3.25.1 | a.104.1.1 | Cytochrome P450 | 0.974 | Metabolism | Oxidation/reduction | ABEV |
| DS.HH.4.26.3 | a.211.1.1 | HD domain | 0.094 | Metabolism | Other enzymes | ABEV |
| DS.HH.4.50.1 | a.25.1.2 | Ribonucleotide reductase-like | 0.485 | Intracellular processes | Inorganic ion transport and metabolism | ABEV |
| DS.HH.4.70.1 | a.211.1.2 | PDEase | 0.863 | Metabolism | Other enzymes | ABEV |
| DS.HH.4.8.4 | a.102.1.2 | Cellulases catalytic domain | 0.378 | Metabolism | Carbohydrate transport and metabolism | ABEV |
| DS.GE.3.21.2 | g.35.1.1 | HIPIP (high-potential iron protein) | 0.485 | Metabolism | Electron transfer/transport | ABV |
| DS.EG.2.16.6 | c.66.1.19 | Histamine methyltransferase | 0.588 | Metabolism | Transferases | BE |
| DS.HH.5.10.3 | a.123.1.1 | Nuclear receptor ligand-binding domain | 0.880 | Regulation | Signal transduction | BE |
| DS.BK.3.56.2 | b.22.1.1 | TNF-like | 0.858 | Extracellular processes | Immune response | BEV |
| DS.EG.1.6.14 | a.119.1.2 | Animal lipoxygenases | 0.678 | Metabolism | Coenzyme metabolism | BEV |
| DS.EH.1.5.32 | c.141.1.1 | Glycerate kinase I | 0.348 | Metabolism | Other enzymes | BEV |
| DS.HH.9.11.1 | d.42.1.1 | BTB/POZ domain | 0.970 | General | Protein–protein interaction (dimerization domains) | BEV |
| DS.BK.4.96.1 | b.2.5.7 | DNA-binding domain from NDT80 | 0.704 | Regulation | DNA-binding (transcription factors) | E |
| DS.HH.5.17.3 | a.7.5.1 | Tubulin chaperone cofactor A | 0.678 | Intracellular processes | Posttranslational modification, protein turnover, chaperones | E |
