# Supplementary figures and images for: Evolution of Intrinsic Disorder in Protein Loops
Source: Life (Basel). 2023 Oct 14;13(10):2055. doi: 10.3390/life13102055 (PMC10608553; doi:10.3390/life13102055)

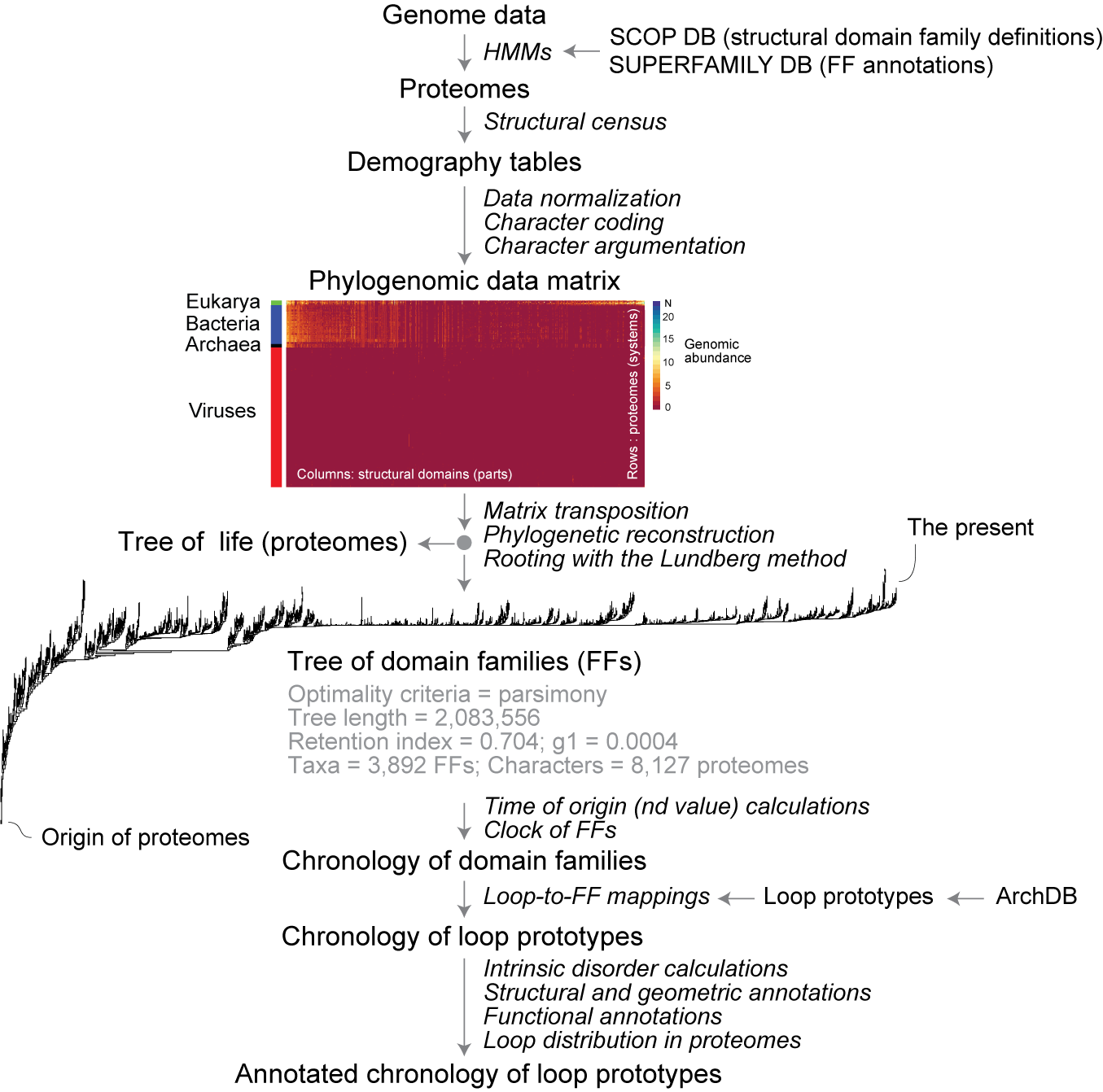

Supplement: Supplementary file 1 [file life-13-02055-s001.zip › Figure S1.tif]
